# Entrepreneurship and Family Role: A Systematic Review of a Growing Research

**DOI:** 10.3389/fpsyg.2019.02939

**Published:** 2020-01-10

**Authors:** Giuseppina Maria Cardella, Brizeida Raquel Hernández-Sánchez, José Carlos Sánchez García

**Affiliations:** Department of Social Psychology and Anthropology, University of Salamanca, Salamanca, Spain

**Keywords:** entrepreneur, family support, parent role, literature review, role models

## Abstract

In recent years, research on the family role and entrepreneurship has increased noticeably, consolidating itself as a valid and current subject of study. This paper presents a systematic analysis of academic research, applying bibliometric indicators, and cluster analysis, which define the state of research about the relationship between family role and entrepreneurship. For this purpose, using three well-accepted databases among the research community: Scopus, Web of Science, Business Source, a total of 92 articles were selected and analyzed, published between 1989 and 2019 (until March). A cluster analysis shows five main areas of literature development: (1) cultural dimension and geneder issue; (2) family business and succession; (3) parental role models and entrepreneurial intentions; (4) entrepreneurship and self-employment; (5) family support and women entrepreneurs. Findings also show how this is a relatively recent field of study, with a multidisciplinary character.

## Introduction

Entrepreneurship is a determining factor of economic development (Thurik, 2009; Hessels and van Stel, 2011; Audretsch et al., 2015), social and structural change (Acs et al., 1999; North, 2005). Entrepreneurship not only contributes to the economic and social growth of a nation, but also stimulates the development of knowledge (Shane, 2000), technological change (Acs and Varga, 2005), competitiveness and innovation (Parker, 2009; Blanco-González et al., 2015). In fact, the European community has promoted numerous actions aimed to improve and develop the entrepreneurial attitude of European citizens toward Business venture, focusing on aspects that are essential for creating a corporate identity. However, the levels of entrepreneurial activity in some European countries are still low. According to the latest international study of Global Entrepreneurship Monitor (GEM), published in 2018, Europe has the lowest TEA (Total Entrepreneurial Activity) of all regions in all age studied. This is a concerning result, especially in it's current crisis period.

Entrepreneurial activity is not just about discovering new ideas and possibilities (Shane and Venkataraman, 2000), but also intentional planning, developed through the cognitive processing of internal and external factors (Del Giudice et al., 2014). Intention is a cognitive process that precedes the effective involvement of the individual in any type of activity (Liñán and Chen, 2009), and in particular, entrepreneurial intention is closely linked to business world (Moriano et al., 2012) and has become a rapidly evolving research sector in the international scene (Liñán and Fayolle, 2015).

Currently, in the literature there are two different theoretical approaches which attempt to clarify why some individuals are more inclined toward an entrepreneurial career when compared to others: the first analyzes personality traits (Zhao and Seibert, 2006; Rauch and Frese, 2007; Leutner et al., 2014; DeNisi, 2015), the second focuses on environmental and behavioral factors (Peterson, 1980; Aldrich, 1990; Baum et al., 2001). Specifically, researchers study the importance of some individual traits as factors predetermining to perform entrepreneurial activities such as high levels of self-efficacy (Krueger et al., 2000; Zhao et al., 2005; Lee et al., 2011; Rasul et al., 2017), risk propensity (Schwartz and Whistler, 2009; Tumasjan and Braun, 2012; Yurtkoru et al., 2014), tolerance to ambiguity, and uncertainty (Hmieleski and Corbett, 2006; Schwartz and Whistler, 2009; Arrighetti et al., 2012), metacognitive abilities and individual abilities (Kor et al., 2007; Dickson et al., 2008; Liñán et al., 2011), locus of control (Battistelli, 2001; Gordini, 2013), as well as creativity (Hamidi et al., 2008; Smith et al., 2016; Biraglia and Kadile, 2017); the environmental and behavioral focuses refers to the Social Learning Theory (Bandura, 1986), according to which, individuals learn certain skills from other people, which act as models. Specifically, the term “role model” emphasizes the individual's tendency to identify with other people occupying important social and the consequent cognitive interdependence of skills and behavior patterns (Gibson, 2004).

In this scenario, the role of the family in guiding young people toward choosing an autonomous/entrepreneurial job becomes important (Fraccaroli and Vitali, 2001; Odoardi, 2003); the social network is an important intangible resource for the development of their business activities (Presutti et al., 2011); in particular, the perception of the family support influences, in the university students, the choice of career in general (Henderson and Robertson, 2000) and specifically the business one (Türker et al., 2005; Taormina and Lao, 2007; Zellweger et al., 2011; Laspita et al., 2012).

This evidence is not enough proof. For example, some researchers have not found a statistically significant relationship between entrepreneurial parenting role models and children's decision to choose an entrepreneurial career (Rodriguez et al., 1999; Kim et al., 2006), other studies, instead, have found a negative effect, especially in situations of failure of the family business (Scherer et al., 1989; Mungai and Velamuri, 2011).

Taking into consideration the ideas exposed above, we conducted this systematic review to analyze the relationship between the role of the family and the entrepreneurial process. Specifically, we aim to answer the following questions:

1. What is the temporal development of research on the relationship of the role model in entrepreneurship?
2. Who are the most productive authors, countries and journals?
3. What are the thematic areas that have been most studied by researchers?

Furthermore, to reduce the risk of bias to a minimum, we applied a series of bibliometric indicators. Bibliometric indicators are defined as a rigorous set of statistical and mathematics methods to be applied to documents and other patterns of knowledge (Pritchard, 1969). It is a method widely used in the literature as it provides an overview of academic research, through the identification of the main trends in a given field of study (Martínez-López et al., 2018). Many bibliometric revisions regarding entrepreneurship have been conducted (Cabeza-Ramírez et al., 2017; Baier-Fuentes et al., 2019). However, specifically to the relationship between family role and entrepreneurship, our research did not generate any results. The only existing revisions take into consideration the family, understood as a family business (López-Fernández et al., 2015).

In the following section we explain the methodology for systematic analysis, and we will report the main results. In the final part, we present the conclusions that can be drawn from our analysis, its limitations, as well as reflections for future developments.

## Materials and Methods

In this article, we review the literature on the family role in entrepreneurial capacity using the systematic analysis method as “explicit, rigorous and transparent methodology” (Greenhalgh et al., 2004, p. 582). In this sense, we collected the publications until March 2019 and extracted the most relevant results, through the application of statistical methods.

To reduce the risk of bias, during the selection phase of the articles, we used a mechanism established in the literature, the PRISMA method (Liberati et al., 2009; Urrútia and Bonfill, 2010), which allows to make the work replicable (Lourenço and Jones, 2006; Pittaway and Cope, 2007).

In order to search for relevant articles, we used three databases: Scopus, Web of Science and Business Source, without defining a specific publication period. The selection of these items was made on basis that are considered the most powerful databases in existence. Specifically, Web of Science because it is the oldest database of citations, dating back to 1900, and provides strong coverage in international research (Li et al., 2010), guaranteeing the highest quality; Scopus, on the other hand, with 27 million abstracts, is the largest database of scientific literature (Burnham, 2006); Business Source as a third database, as it provides a repertoire on entrepreneurial, business, and economics sciences literature.

Initially, within each database, we applied the Boolean search terms “famil^*^ role,” “famil^*^ support,” “parent^*^ role,” “parent^*^ support,” and “entrepren^*^” to identify all the publications that contained the keywords in the title of publications, author of key words or abstract. After eliminating all the duplicate articles, a total of 192 documents were identified over a period of time between 1989 and 2019. All 192 abstracts were read to ensure that the document deals with our construct. When a doubt arose, the entire document was read to confirm this.

As far as the inclusion/exclusion criterion is concerned, we have only considered journal articles since they are scientific knowledge (Podsakoff et al., 2005), written in English or Spanish language, and containing a direct relationship between the family (parental) role and entrepreneurship. On the contrary, were excluded: chapters of books and conference papers, publications that did not make any connection between the two constructs, or that analyzed the role of family members other than the parental couple (for example, possible partners or brothers), and all articles written in a language other than English or Spanish. For example, we have excluded the article by Fernández Robin et al. (2017) because they mention “the role of the family” in the abstract, but they refer specifically to housewives for women and how entrepreneurship and of family assistance seem incompatible, or the article by Logan (2014), as it analyzes the relationship between family and entrepreneurship, but refers to the support received from the partner or spouse.

A total of 92 articles were analyzed in this study (Annex 1 in Supplementary Material).

**Figure 1:**
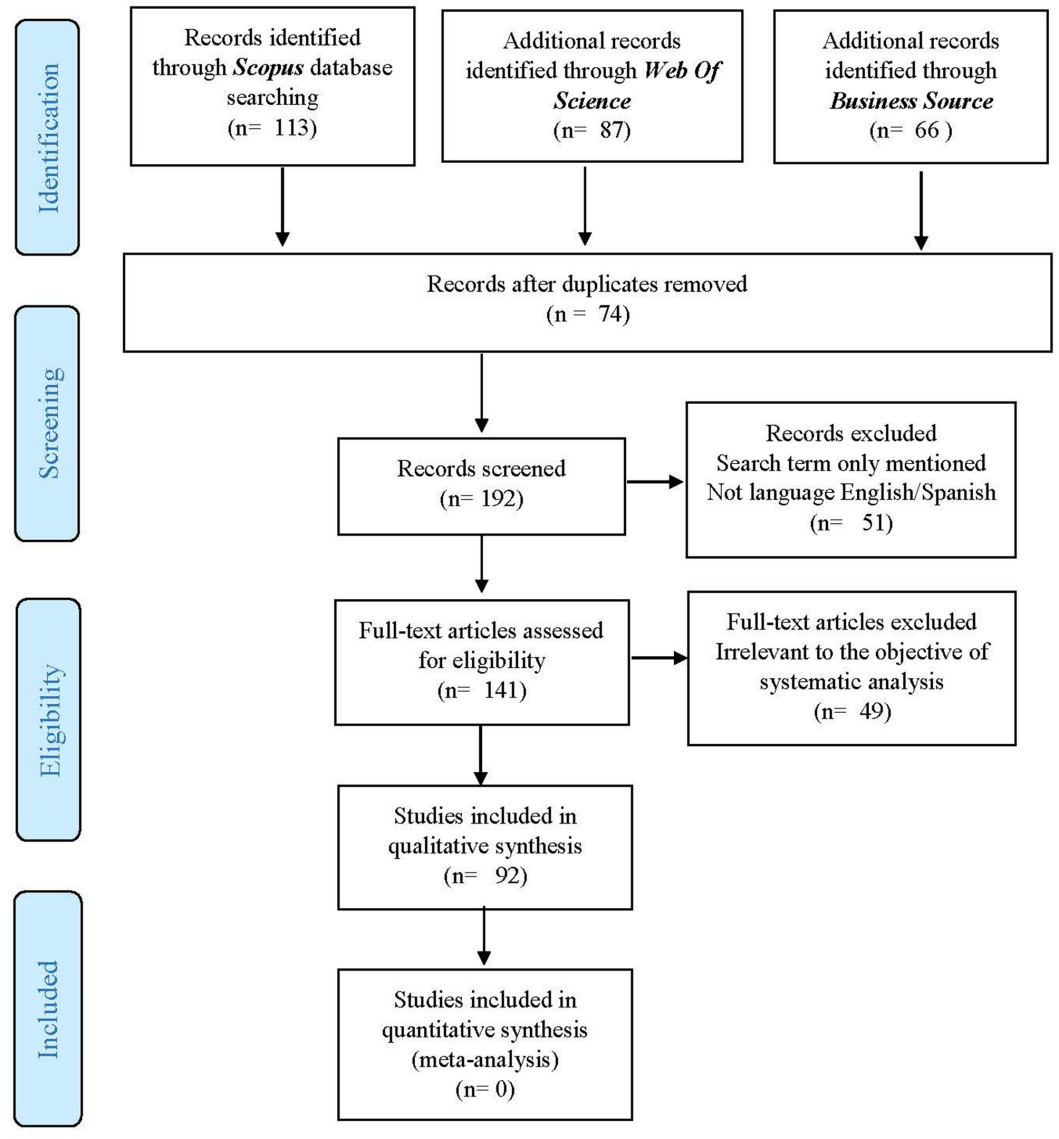
PRISMA 2009 flow diagram.

Figure 1 shows the flow Diagram of the study according to the recommendations of the PRISMA method.

Different types of indicators have been used.

Specifically, we analyzed year of publication, the productivity of authors, countries and journals, research area (e.g., Social Science, Business and Management, Economic etc.), type of research and sample. In addition, in order to measure the impact on productivity and on citation of an author's publications and journals we used the *h*-index (Hirsch, 2005). In this sense, as underlined by Gaviria-Marin et al. (2018), it is an important bibliometric indicator that is commonly used by researchers given its ease of interpretation.

To analyze the most investigated thematic areas, was used the analysis of the co-occurrence of the authors' keywords, through the VOSviewer software version 1.6.10 (Van Eck and Waltman, 2010, 2014). It is a bibliometric technique that allows graphic representation, identification and classification of clusters in a strategic matrix associated on the basis of similarities and dissimilarities (distance-based maps). Moreover, while the qualitative analysis of the literature can be affected by the subjectivity of the author, this method allows to overcome this problem, becoming an instrument of undisputed and consolidated analysis (Vallaster et al., 2019), used in presently (Valenzuela et al., 2017; Martínez-López et al., 2018).

In Table 1 we show a summary of the main methodological features of the study.

**Table 1:** Characteristics of the bibliometric study.

Search terms	“famil* role”; “famil* support”; “parent* role”; “parent* support” AND “entrepren*”	
Mentioned at least once in	Abstract, Title, or Keyword (Scopus) Topic or Title (Web of Science)	
Time period	1989–2019	
Language	English or Spanish	
Document type	Peer-Reviewed Articles	
Primary database		Records
	Scopus Web of Science	113 87
Secondary database (quality checks)	Business Source	66
Total articles		266
Records after reading all abstracts to ensure that all articles were related to the search object (Excluding duplicates and no Peer-Reviewed Articles)		192
Final analyzed records		92
**Analysis tools (bibliometric indicator)**	Quantitative analysis (Spss Statistics 0.25); *h*-index and Cluster Analysis (VOSviewer)	

## Results

**Figure 2:**
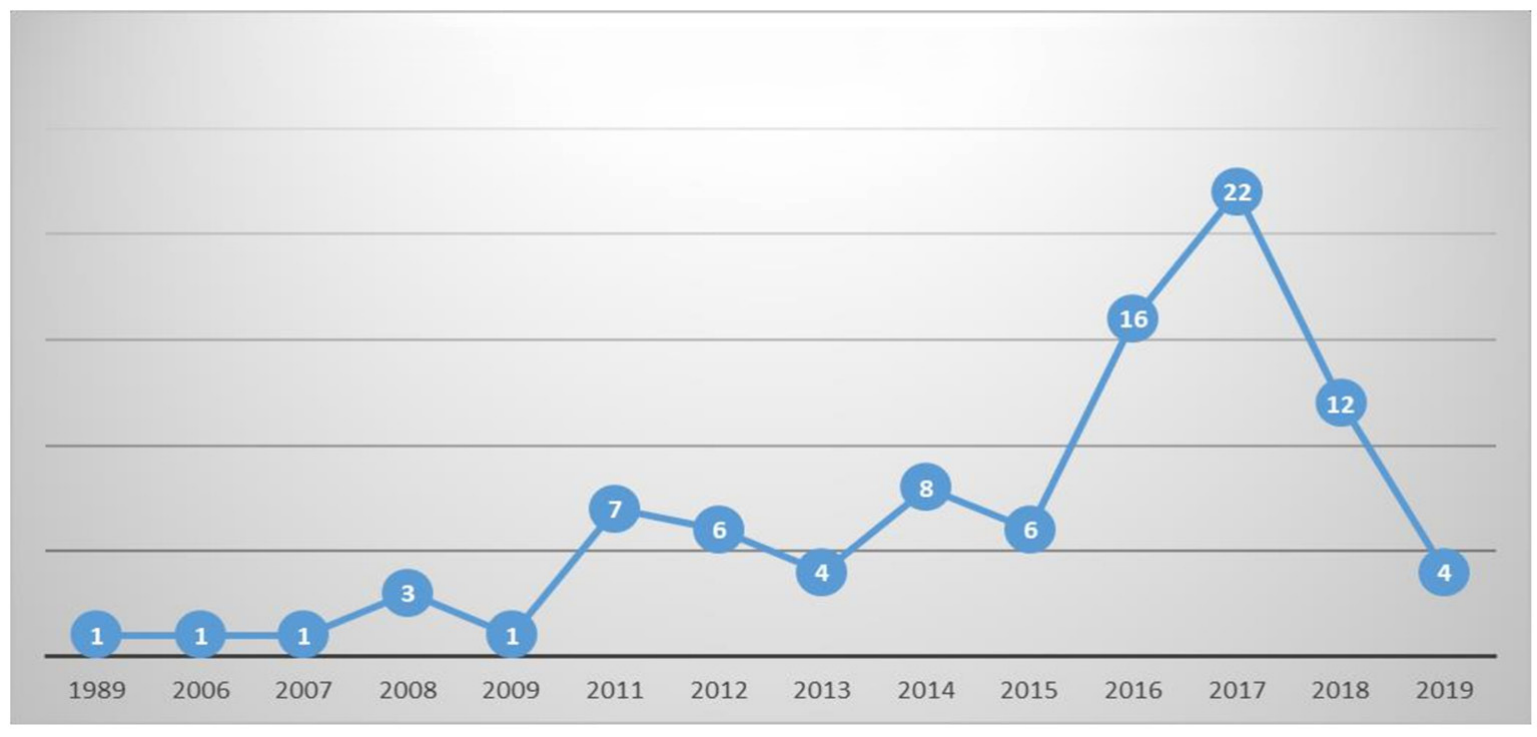
Evolution of publications.

Figure 2 illustrates the growth, during the period 1989–2019, of the family role and entrepreneurship publications in the international scene. Research has experienced great development in recent years, in fact, since 2011, the interest in topics concerning the relationship between family and entrepreneurship has increased significantly, recording the most profitable peak of publications in 2017. Although only the first 3 months of 2019 are included in the data set, 4 articles had already been published during this period.

This increase in publications in recent years may suggest a continuous evolution of family role in entrepreneurship as current and still valid research trend topic.

**Figure 3:**
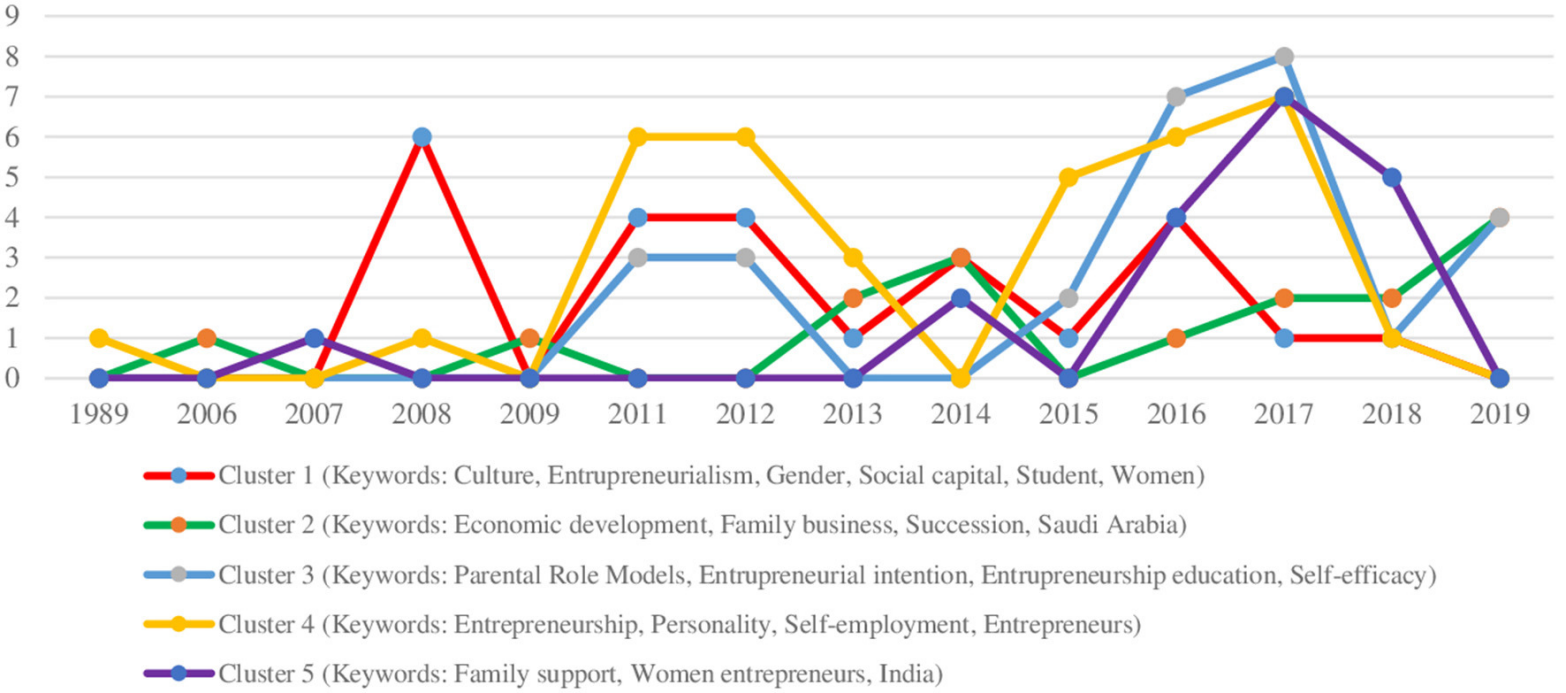
Article published by cluster by year.

In order to analyze the trend of research in the family/parent support and entrepreneurship constructs, we used the dimensions obtained from cluster analysis. Figure 3 shows the progress of the research from 1989 to 2019. As noted, the constructs are associated with different fields of research, emphasizing the multidisciplinary character.

For example, over the years, the trend of research interests has changed. From the interest shown by the literature toward the cultural and social dimension (cluster 1—red line) and toward the individual personological characteristics of the entrepreneur (cluster 4—yellow line), in recent years the dimension that has received the most attention is the family one. The two most in-depth research themes, with a peak of interest between 2017 and 2018, are: the influence of parental role models and the educational dimension in the development of entrepreneurial intentions (cluster 3—turquoise line), and the importance of family support (cluster 5—purple line), especially in developing countries and disadvantaged groups, such as female entrepreneurship.

Furthermore, in the year 2019 it would seem that there is a potential recovery for the theme of family businesses as factors of economic development, but clusters 2 and 3 would still seem to be actual.

Afterwards, we analyzed the productivity of scientific journals, generating a list of 92 articles. For the purposes of our analysis, we have considered journals with a minimum of 3 publications on the subject, classifying them from the most productive to the least productive. As can be seen from Table 2, the scientific journal that has more active the role of the family in the entrepreneurial process is International Journal of Entrepreneurial Behavior and Research (*n* = 6 articles; *h*-index = 44).

**Table 2:** Article with the most publications on the subject.

**No**.	**Journals**	***h*-index**	**Research area**
6	Int. J. Entrep. Behav. Res.	44	Business and Management
4	Journal of Business Research	166	Business and Management
4	International Journal Gender and Entrepreneurship	–	Social science
4	Journal of Entrepreneurship	15	Business and Management
4	Small Business Economics	108	Economics
3	Academy of Entrepreneurship Journal	–	Business and Management
3	Entrepreneurship: Theory & Practice	128	Business and Management
3	Int. J. Entrepreneurship and Small Business	–	Business and Management
3	Journ. Small Business and Enterprise Development	–	Business and Management

The analysis also revealed the multidisciplinary nature of the research area. Most publications (*n* = 71) are related to business and management research, but others come from psychology and social sciences (*n* = 14), economics (*n* = 6), and engineering (*n* = 1).

We performed as well an analysis to identify the authors who, are considered most influential in the development of this field of study. In the 92 articles that were part of the bibliometric study, a total of 239 authors were found (2.59 authors per article). 90.9% contributed with only one work on the subject, which shows that it is a highly dispersed field, probably due to its multidisciplinary nature.

This interpretation gained more strength after verifying that only 8 authors participated in two or more articles, as shown in Table 3. The first 3 authors with 4 articles are Kaciak, E. (h-index = 8); Memili, E. (h-index = 13), and Welsh, D. (h-index = 14).

**Table 3:** Authors with the greatest number of articles published.

**No**.	**Author**	**Country**	***h*-index**	**Affiliation**	**Main subject**
4	Kaciak, E.	Poland	8	Kozminski University	Economic development; women entrepreneurs
4	Memili, E.	USA	13	The University of North Carolina	Family business; New venture creation
4	Welsh, Dianne H.B.	USA	14	The University of North Carolina	Economic development; Family business; Women entrepreneurship
2	Bignotti, A.	South Africa	1	University of Pretoria	Entrepreneurship education;
2	Le Roux, I.	South Africa	2	University of Pretoria	Contextual variables; Entrepreneurial endowment; Personality traits; Youth entrepreneurship
2	Khan, Muhammad	Saudi Arabia	1	Effat University	Entrepreneurship ecosystem; Female Start-ups; Saudi Arabia; Success factors
2	Morales-Alonso, G.	Spain	6	Universidad Politécnica de Madrid	Entrepreneurial intention; Parental role models; Attitudes toward work
2	Pablo-Lerchundi, I	Spain	2	Universidad Politécnica de Madrid	

**Figure 4:**
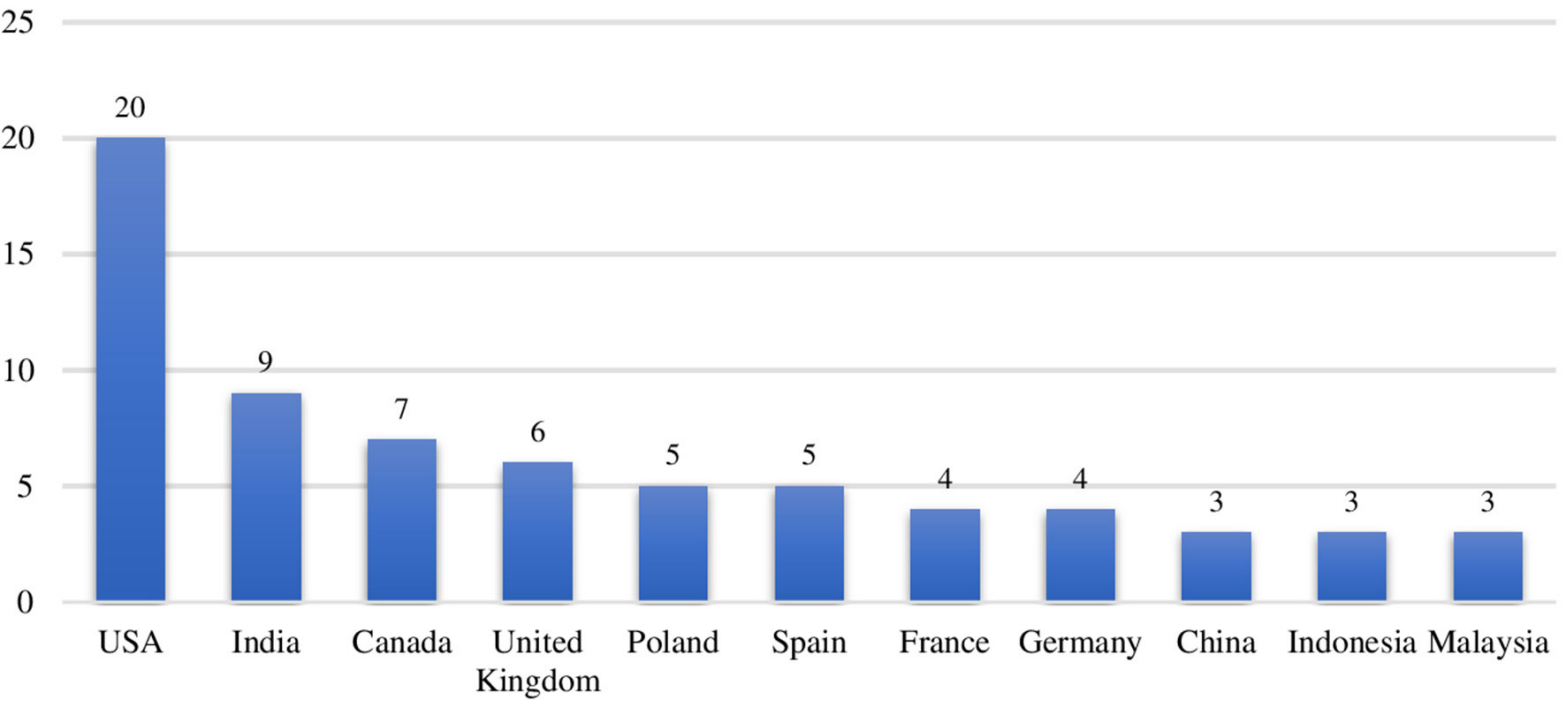
Publications of countries.

Were also analyzed the countries where the research field of our object of study is more developed (Figure 4). Therefore, for the purposes of this analysis we have considered only countries with a minimum of 3 publications. The United States is the country with the largest number of publications (*n* = 20), followed by India (*n* = 9), and Canada (*n* = 7). The United Kingdom (*n* = 6) and Spain (*n* = 5) follow, in fourth and fifth place of the rank, and represent the two most productive countries in Europe in terms of research on the role of the family and entrepreneurship.

Finally, we conducted an analysis on the nature of the research and the type of sample. The quantitative analysis is the most used in the selected studies (69.6%), using a variety of analysis techniques: descriptive (*n* = 34) logistic, linear and hierarchical regression (*n* = 22); confirmatory, using Structural Equation Modeling (*n* = 14); correlation (*n* = 17); *t*-test (*n* = 6); univariate (*n* = 5); and multivariate (*n* = 4). Qualitative studies (*n* = 19), on the other hand, the ones less frequently used are: observation (*n* = 3), case studies (*n* = 5), interviews (*n* = 6), in-depth interviews (*n* = 8), and focus group (*n* = 1), representing only 20.7% of the studies. Most of the articles applied more than one analysis technique. Finally, four articles (4.4%) used a mixed method (quantitative and qualitative research).

The results are summarized in Table 4.

**Table 4:** Nature of research and type of sample.

**Nature of research**	**Total**	**% of the sample**	**Type of sample**	**Total**	**% of the sample**
Quantitative	64	69.6%	Entrepreneurs	34	37%
			Students	27	29.4%
Qualitative	19	20.7%	Entrepreneurs	16	17.4%
			Students	1	1.1%
Review	1	1.1%	Entrepreneurs	1	1.1%
Mixed	4	4.4%	Entrepreneurs	4	4.4%
No Empirical	3	3.3%			

Descriptive statistics and regression analysis are the most commonly used techniques in the reviewed articles, followed by correlation analysis and confirmation analysis through Structural Equation Modeling. The latter was mostly used, especially in more recent articles.

As for the type of sample used, the studies with entrepreneurs prevailed in 59.9% of the analyzed articles (of which 26.7% were female entrepreneurs), while the studies that analyzed students accounted for 30.5%. 7.7% of the studies considered other types of samples that do not fall into the categories previously explained.

En general, to identify the state of research on the relationship between family role and entrepreneurship, proceeded the co-occurrence analysis with one occurrence per keyword, for a total frequency of 237 authors' keywords grouped in 25 clusters.

**Figure 5:**
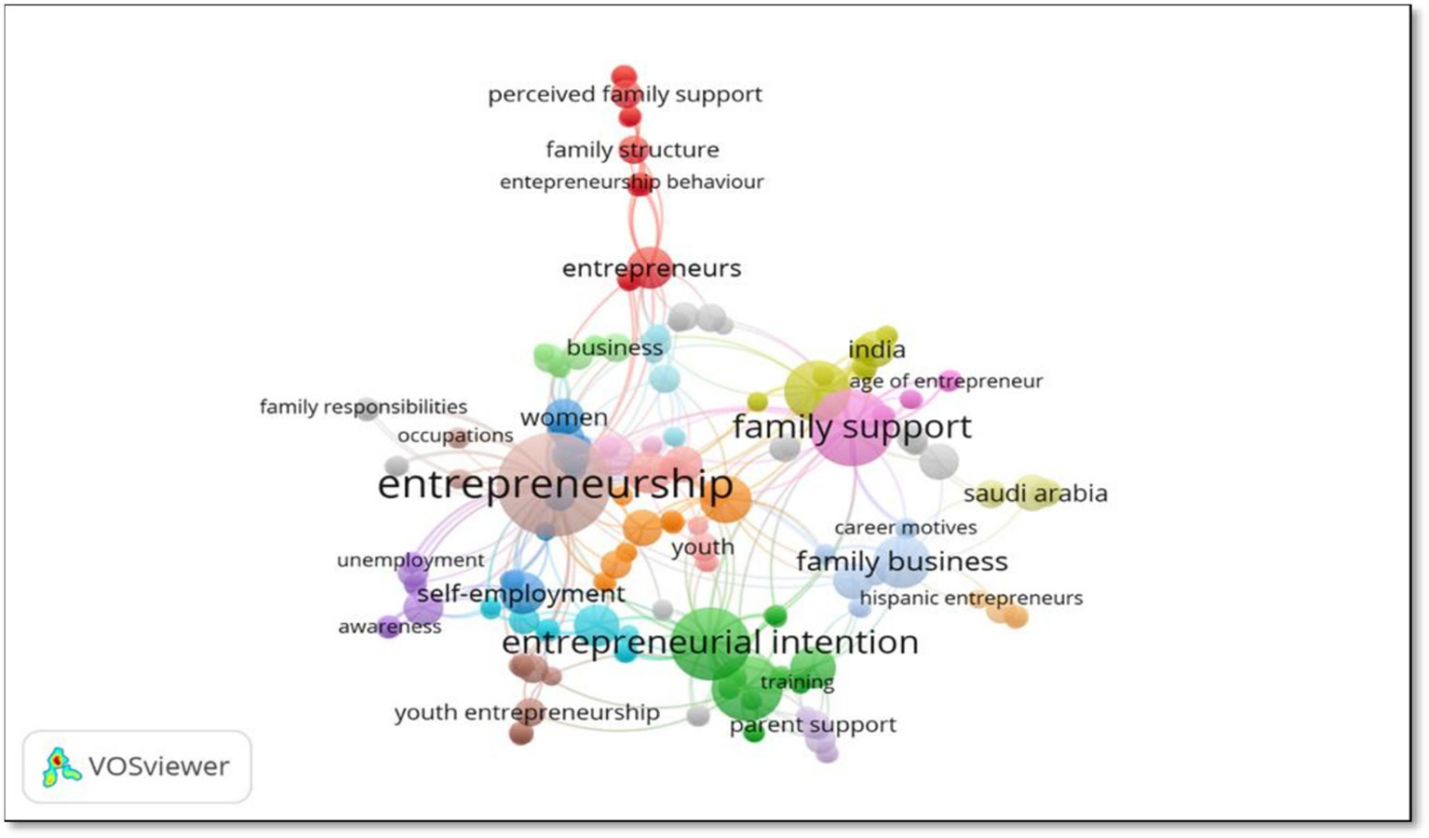
One co-occurrence for keywords. Source: VOSviewer version 1.6.10.

As shown in Figure 5, the stronger relationships are graphically represented by larger circles and labels. The research topics most closely examined by scholars deals with entrepreneurship, family support and entrepreneurial intent.

**Figure 6:**
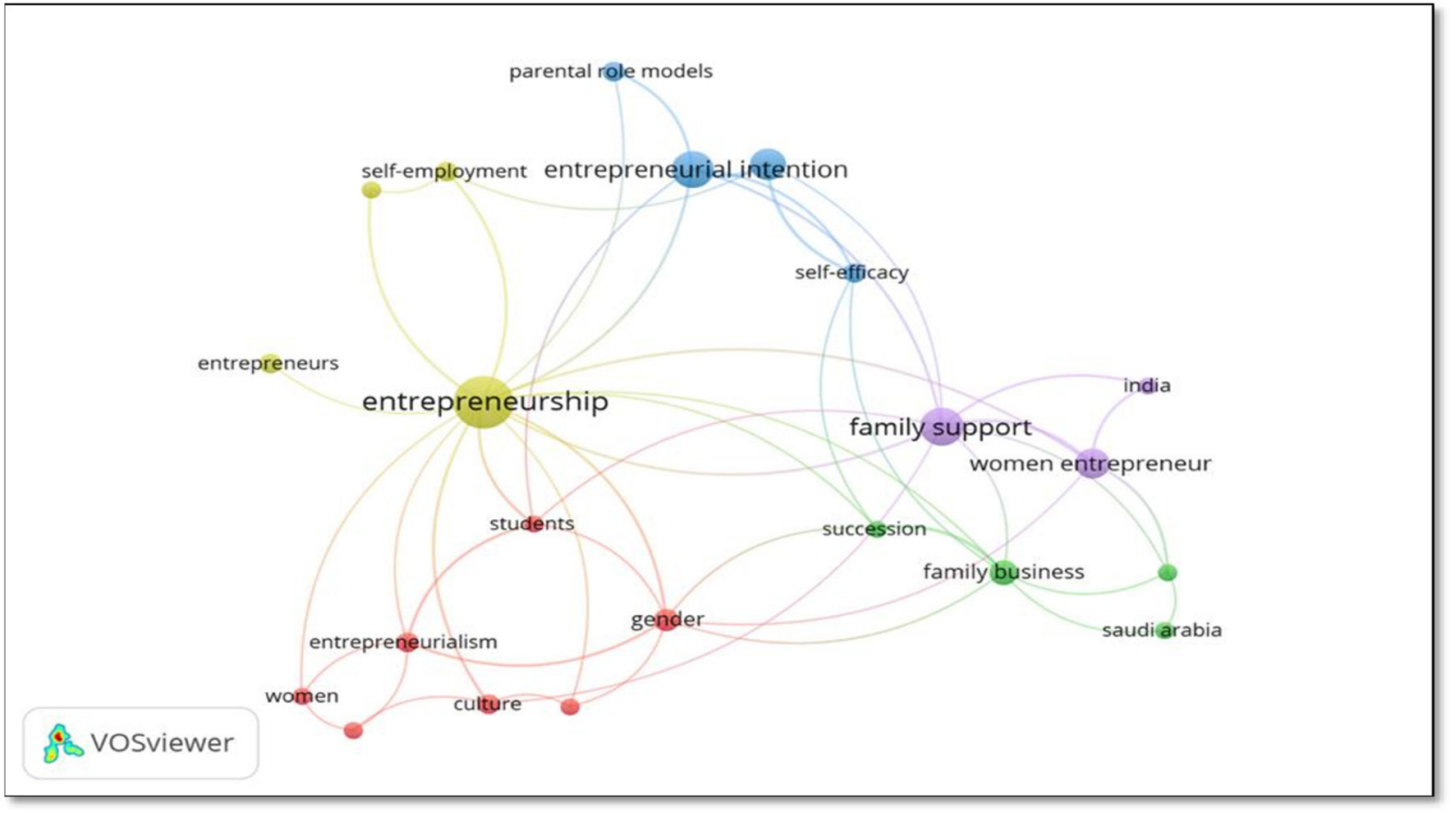
Three co-occurrence for keywords. Source: VOSviewer version 1.6.10.

For the purposes of the study, we have narrowed the field, performing a co-occurrence analysis with a minimum of three occurrences for keyword, for a total of 22 authors' keywords. The mapping and grouping provides a general review of the research in the context of entrepreneurial literature and in Figure 6 are shown the five most relevant clusters. Each cluster is represented by a different color that highlights the relationship between them while the distance between the clusters provides information on the intensity of the relationship (Van Eck and Waltman, 2010).

### Cluster 1: Cultural Dimension and Gender Issue (7 Items)

The occurrence of 21.8% of the keywords studied is associated with the red cluster formed by the following keywords: culture, women, female entrepreneurship, entrepreneurialism, gender, social capital and students.

The cultural dimension is one of the key elements for the family background and entrepreneurial process. According to some authors (Li, 2007; Gurel et al., 2010; Castillo-Palacio et al., 2017) the social and family context in which the individual growths, shapes his creative thinking, predisposes him to innovation and risk perception, develops social capital, generates value, thus creating cultures that encourage more entrepreneurship and autonomy than others.

As suggested by Zhao et al. (2012) there are two different theoretical explanations about the role that culture plays in business world. The first one, of a psychological nature, acting at the individual level (Hayton et al., 2002) and presupposes that culture acts on the skills and abilities of individual, modifying the behavior. The second line, based on institutional theory, considers culture as a substratum of the community, so some societies are more likely to promote entrepreneurial processes.

Several authors use this prospective to analyze entrepreneurial activity in different contexts, for example, Welsh et al. (2018), applying institutional theory, compared women entrepreneurs in Morocco and Turkey. Other studies have focused on other business contexts (McIntosh and Islam, 2010; Ramadani, 2015), predominantly Islamic (Anggadwita et al., 2015; Mohd Rhouse et al., 2016), and Middle Eastern cultures (Bastian et al., 2018).

From this point of view, the cultural dimension is closely related to the “woman” variable and more generally to the “gender issue” because, as several studies have shown (Freytag and Thurik, 2007; Sengupta et al., 2013), behavior is often a consequence of different socio-cultural values that are taught and learned since youth and that last over time, which also applies to entrepreneurial behavior (Hofstede, 2001; Eid, 2006).

Indeed, despite the positive aspects of entrepreneurship understood as a career accessible to all and economically advantageous, a more in-depth analysis shows that there are many cultural obstacles (Ahl and Marlow, 2012), especially for women.

Specifically, the gap between male and female entrepreneurship has been explained by various theories, among which the most exhaustive is the “Social Role Theory” developed by Eagly (1987). According to this explanation, the male group is configured as the ideal for the entrepreneurial field (Bird and Brush, 2002), while women consider entrepreneurship less as a career path (Ahl, 2006).

Rubio-Bañón and Esteban-Lloret (2016) conducted a research to analyze the possible differences between male and female entrepreneurs in 55 different countries, considering cultural factors as among the most relevant hindrances for entrepreneurship (Bosma and Kelley, 2018). The observed results do not yet confirm that cultures with a higher rate of masculinity lead to a greater gender gap in female entrepreneurship rates. Other research has shown that in communities with high virility, women can share and take ownership of these cultural values and be more motivated toward an entrepreneurial career.

Indeed, the relationship between gender and cultural beliefs is still unclear: some studies have shown that women are pursue less an entrepreneurial career (Chen et al., 1998; Gupta et al., 2005). Other studies, instead, suggest that in cultures considered to be stereotypically masculine, women are more inclined toward entrepreneurial activity (Mueller and Conway Dato-on, 2008; Cardozo Crowe, 2010).

The cultural component, as a substrate of a society, comes into play with the variable “students,” in fact, as the literature shows, it is important to adopt policies that support entrepreneurial development at school.

The promotion and enhancement of the “entrepreneurial culture” has become an important component in the initiatives and in the offer of services for students. Universities are called to accept this challenge to prepare students for the acquisition of entrepreneurial skills and competences that allow them to cope with the multiplicity of today's society, in constant evolution and change (Bygrave, 2004).

Promoting student entrepreneurship means making them more aware of their future, in the field of business, to translate ideas into actions.

This cluster shows a relationship with the main terms of the analysis: entrepreneurship and family support, because cultural factors pass through the micro-social dimension of society, including the institutions that live in that community. Social agencies like the school, but also the family, have the task of creating an entrepreneurial-supportive environment that can encourage entrepreneurial activities in students, helping to develop an entrepreneurial culture (Roffe, 1999). Supporting this point of view, many authors (Pruett et al., 2009; Al-Harthi, 2017) agree that regardless of the type of person, different strategies can be used to motivate the students in choosing an entrepreneurial career, encouraging them to work independently and to expose them to entrepreneurial success stories that can serve as models for the acquisition of skills, technical knowledge and relevant know-how.

### Cluster 2: Family Business and Succession (4 Items)

The green cluster consists of the following keywords: family business, succession, economic development, and Arabia Saudita, which constitute the 12.1% of the occurrences.

The authors agree that the factor that distinguishes family and non-family businesses is the intention to transfer the control of the company to its following generation (Chua et al., 1999), a factor that also contributes to economic growth in the developing countries (and also in advanced economies).

The intertwining of family firms and business has a profound impact on entrepreneurial experiences, especially for children, it is so influential that it is considered by Rogoff and Heck (2003), together with human capital and education, as the oxygen that fuels the entrepreneurial fire.

Family businesses are important, not only from a financial point of view, but also because provide long-term stability in the labor market because of the responsibility they show to communities, since they convey values and knowledge. All these factors are valuable instruments of change to counteract the current financial crisis. As highlighted in the final report on family businesses, conducted in 2009 by the European Commission, at European level, more than 60% of existing businesses are family-run. “Most SMEs (especially micro and small enterprises) are family businesses and a large majority of family companies are SMEs” (European Commission, 2009, p. 4).

However, the successor's intention to continue their family's business depends on whether their parents are willing to support them, contributing to the development and success of their family activities (De Massis et al., 2014). In fact, despite the undoubted importance that family succession has from an economic and social point of view, international studies have shown that the newer generation has low interest as well as intention to work in their parents' business (Zellweger, 2017). According to the Sieger et al. (2016), conducted in 50 countries, 8.8% of the 122,000 university students intend to start their own business, but only 2.7% want to be part of the family business. A model of “employee first, then founder” emerges 5 years after studies, in which 38.2% intend to found a business, but only 4.8% consider themselves as employee in their own family business (Sieger et al., 2016). Similar results were achieved by Zellweger et al. (2011), who found that the possibility of being able to inherit the family business does not make it a desirable choice. The successors tend to feel confident about their skills and knowledges, but appear pessimistic about the succession because they considered themselves less autonomous.

In the current context, characterized by an aging population and the desire of many entrepreneurs to transfer the family business to their children, this result is worrying (Garcia et al., 2018).

The performance of those who enter the family business is better when perceived family responsibility as strong, this result highlights the strength of family expectations in positively influencing members' performance (Dawson et al., 2015).

One of the very few studies on the succession of daughters in the family business, conducted by Overbeke et al. (2013), examined the factors that may contribute to this generational shift. The results revealed that family support and leadership tutoring are the most important elements.

Parental support in family businesses is very important not only in the succession phase, but also when the company is consolidated, for example, based on data from 228 entrepreneurs, Marshall et al. (2018) found that the active involvement of the family creates a common destiny among members that favors resilience for an entrepreneur, compared to the owners of non-family businesses.

It is important to understand that the factors that influence the intentions of the members of the next generation to undertake an entrepreneurial career requires a systemic analysis that also considers the behaviors of their parents (Nordqvist and Melin, 2010) and the perception that children have of this support (Garcia et al., 2018).

### Cluster 3: Parental Role Models and Entrepreneurial Intention (4 Items)

The third cluster associates the following keywords: entrepreneurial intention, entrepreneurship education, parental role models and self-efficacy. The 22.6% of keywords are related to this cluster which emphasizes the importance of entrepreneurial education, parental role model and self-efficacy for entrepreneurial intention development.

Historically, intentions have been considered as the antecedent of behavior (Ajzen and Fishbein, 1977; Ajzen, 1991). The meta-analysis by Sheeran (2002) conducted on 422 studies, showed that the correlation between intentions and behavior explains 28% of the variance in behavior. For this reason, much of the literature has been interested in studying the factors influencing intentions. In this regard, in recent decades, great importance has been attached to the positive influence played by role models in improving the intentions of choosing an entrepreneurial career. Bosma et al. (2012) found that 54% of a sample of 292 entrepreneurs had a role model (20% in the pre-start-up phase, 10% in the post-start-up phase and 24% in both phases), in addition, one-third of the sample stated that they would not have founded their company without this role model.

The positive influence of role models on entrepreneurial intentions has been empirically analyzed in various cultural contexts. A German study by Chlosta et al. (2012) showed that parental role models increased the likelihood of individuals becoming self-employed. Urbano et al. (2011), instead, established that individuals with the same ethnicity can act as a model, encouraging other individuals in the community to create new businesses. The study conducted by Pablo-Lerchundi et al. (2015) showed that the profession carried out by parents influences the entrepreneurial intentions of students, who were more likely to choose an entrepreneurial career if their parents were entrepreneurs than children of public officials. In recent years, the impact of role models on entrepreneurial process was confirmed in different professional categories, as in academic entrepreneurs (Fernández-Pérez et al., 2015) and active entrepreneurs (Bosma et al., 2012; Fritsch et al., 2012).

Self-efficacy has also been considered an important factor that increases the intentions to undertake a certain behavior, especially if associated with a positive attitude toward this behavior (Markham et al., 2002). Relationship between self-efficacy and parental role models as well as attitudes toward entrepreneurship have been established in numerous studies. For instance, Carr and Sequeira (2007) in a research conducted on 308 individuals, found direct and indirect effects of previous family exposures on entrepreneurial intention, through the mediation of perceived family support and entrepreneurial self-efficacy. BarNir et al. (2011), which indicated the positive influence of role models on entrepreneurial intention and the role of mediation exercised by self-efficacy, arrived at the same conclusion. Similar studies were conducted for female university students (Sahinidis et al., 2019). Laviolette et al. (2012) found that role models positively influence entrepreneurial intentions by increasing self-efficacy, provided that such models are positively perceived by individuals, so as to enable them to identify themselves.

Furthermore, role models also play a key role in entrepreneurial training processes, positively influencing the development of entrepreneurial skills (Heinonen and Poikkijoki, 2006). Entrepreneurship education, effectively, influences on the intention of undertaking autonomous activities through two objectives: creating and spreading knowledge (Perreira and Da Silva, 2003) and encouraging students to develop skills in human capital (Gupta and York, 2008). The importance given to the role of education in the entrepreneurial process is underlined by the Global Entrepreneurship Monitor (GEM) which dedicated the special theme of 2008 to Entrepreneurship Education and Training.

In the literature there are studies that explain how perceived family support can come into play in this process. For example, in a research by Denanyoh et al. (2015) emerged that university support, structural support and emotional support of the family are important factors that influence the entrepreneurial intention of students in Ghana. The same result emerges from a study conducted by Bignotti and le Roux (2016) which found that entrepreneurship education and family support positively influence students' need for achievement and entrepreneurial intentions. In another study conducted, Laguía et al. (2019) found that the perceived family support and university support are positively associated with entrepreneurial intentions in students. Furthermore, entrepreneurial self-efficacy and entrepreneurial education moderate the relationship between support and entrepreneurial intention.

At the same time, the research emphasized the importance of entrepreneurship education as a possible tool that, based on skills and knowledge useful to the subjects in order to achieve greater self-confidence and security, could lead to overcoming the gap between men and women in the entrepreneurial field (gender bias).

Entrialgo and Iglesias (2017), on a sample of 338 students found that the role models and entrepreneurship education have a greater positive influence on attitudes toward entrepreneurship in women compared to men.

Exposure to parental role models and entrepreneurship education can be used as tools to reduce the negative prejudicial effects, in general and those related to female entrepreneurship in particular, improving attitudes toward an autonomous career choice.

### Cluster 4: Entrepreneurship and Self-Employment (4 Items)

**Figure 7:**
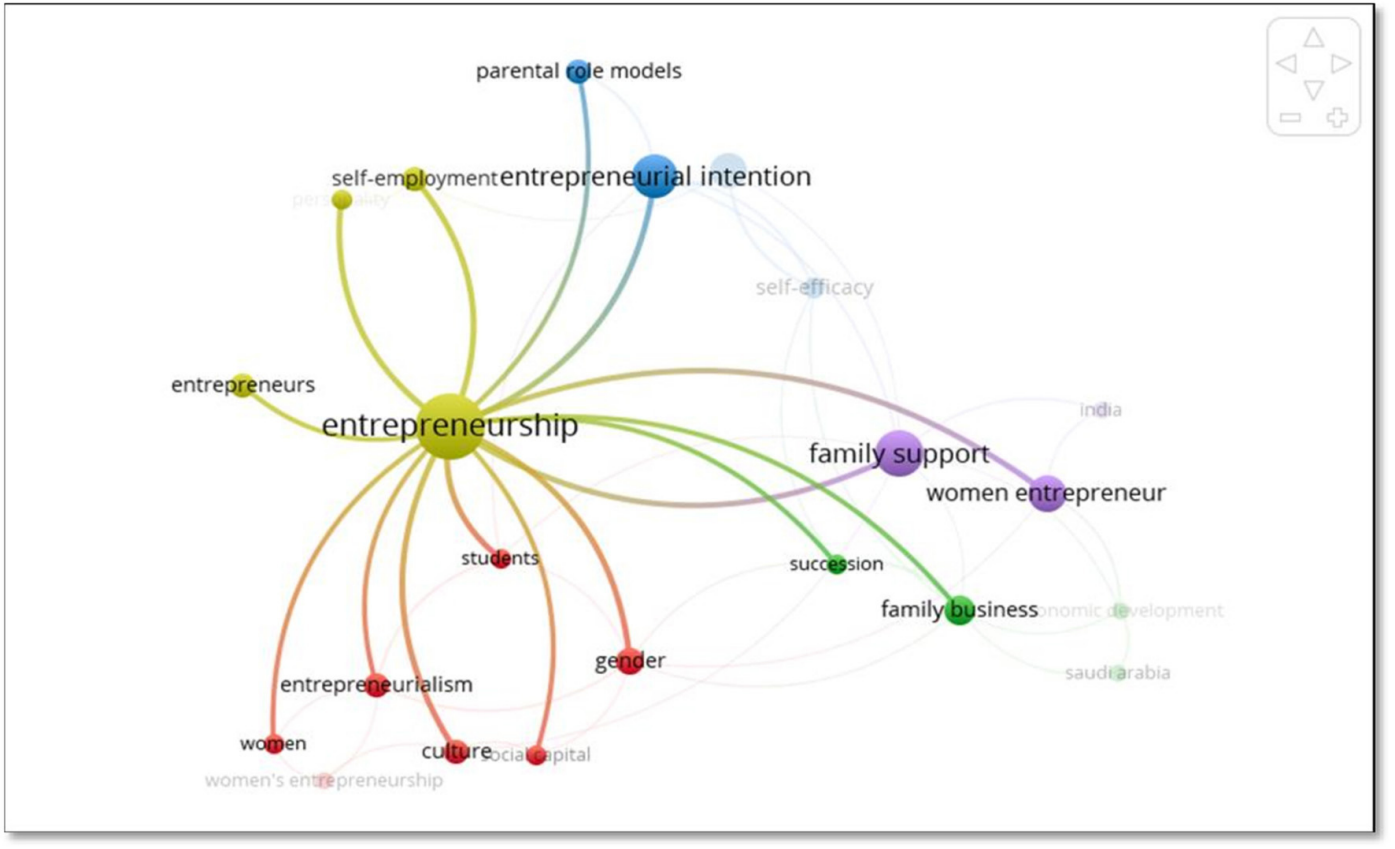
Relations of the yellow cluster. Source: VOSviewer version 1.6.10.

The co-occurrence of 25% of keywords is related to the fourth cluster that shows the greatest number of connections in the map. The following words are part of this cluster: entrepreneurship, self-employment, entrepreneurs, and personalities (Figure 7).

Entrepreneurship is considered instrumental to economic growth and technological development (Fellnhofer and Kraus, 2015; Nowinski and Haddoud, 2019), as an important source of employment in developed and developing countries (Kuratko, 2005). It is not just a factor of economic growth aimed at creating new jobs; it also constitutes a useful personal development tool contributing to the resolution of social issues by promoting a society capable of attributing the correct value to the entrepreneurial mind, and by fostering development of positive attitudes in achieving objectives that concern the community. For example, according to The European Commission (2003), it is a state of mind and a process aimed at creating and developing economic activity by combining willingness to take risks, creativity and innovation.

For the reasons mentioned above, discovering which factors, at the micro and macro level, can lead people to pursue an entrepreneurial career, in recent decades has been the one of the central theme of scholars. In particular, studies conducted on the characteristics of potential entrepreneurs tend to focus, especially on the importance attributed to personality traits (this explains the strong relationship between the words “entrepreneurship” and “personality”), but also, albeit with less strong relationships, to the resources accumulated from education and experience (educational and family background) (Serneels, 2008) and specific behavioral models (Liñán and Fayolle, 2015), which is why, in our analysis, it represents the construct with more relationships with other clusters .

The study of the phenomenon of entrepreneurship can be divided into two phases. In the initial stages of the research, the psychological literature has focused on the study of the personality and the motivations that push a subject to undertake this choice and that can lead to a possible work and personal success (Boyd and Vozikis, 1994). Caird (1993), in an attempt to trace a profile of the typical entrepreneur, offered a synthesis of the results of the researches that have used psychological tests on entrepreneurs, it is necessary to underline that the poor homogeneity of the entrepreneurial population represents a critical aspect for this survey. For this reason, currently, the focus has shifted to the interaction between socio-economic and cultural reality, and decision-making behaviors capable of influencing a chain of events on different levels (personal, family, and economic) (Shane, 2003; Rauch and Frese, 2007). What we are witnessing, in fact, is a decentralization of personal characteristics and a greater attention to complex behaviors acted along different phases of the entrepreneurial process. However, the effects of the cultural-family component have not yet been fully clarified (Ucbasaran et al., 2008). Research on the creation of new businesses has focused mainly on the importance of higher education and employment, with a limited emphasis on education received in the family. This could be the explanation about the challenging why it is so difficult to establish clear links between the role of the family and the potential entrepreneurial spirit.

### Cluster 5: Family Support and Women Entrepreneurs (3 Items)

**Figure 8:**
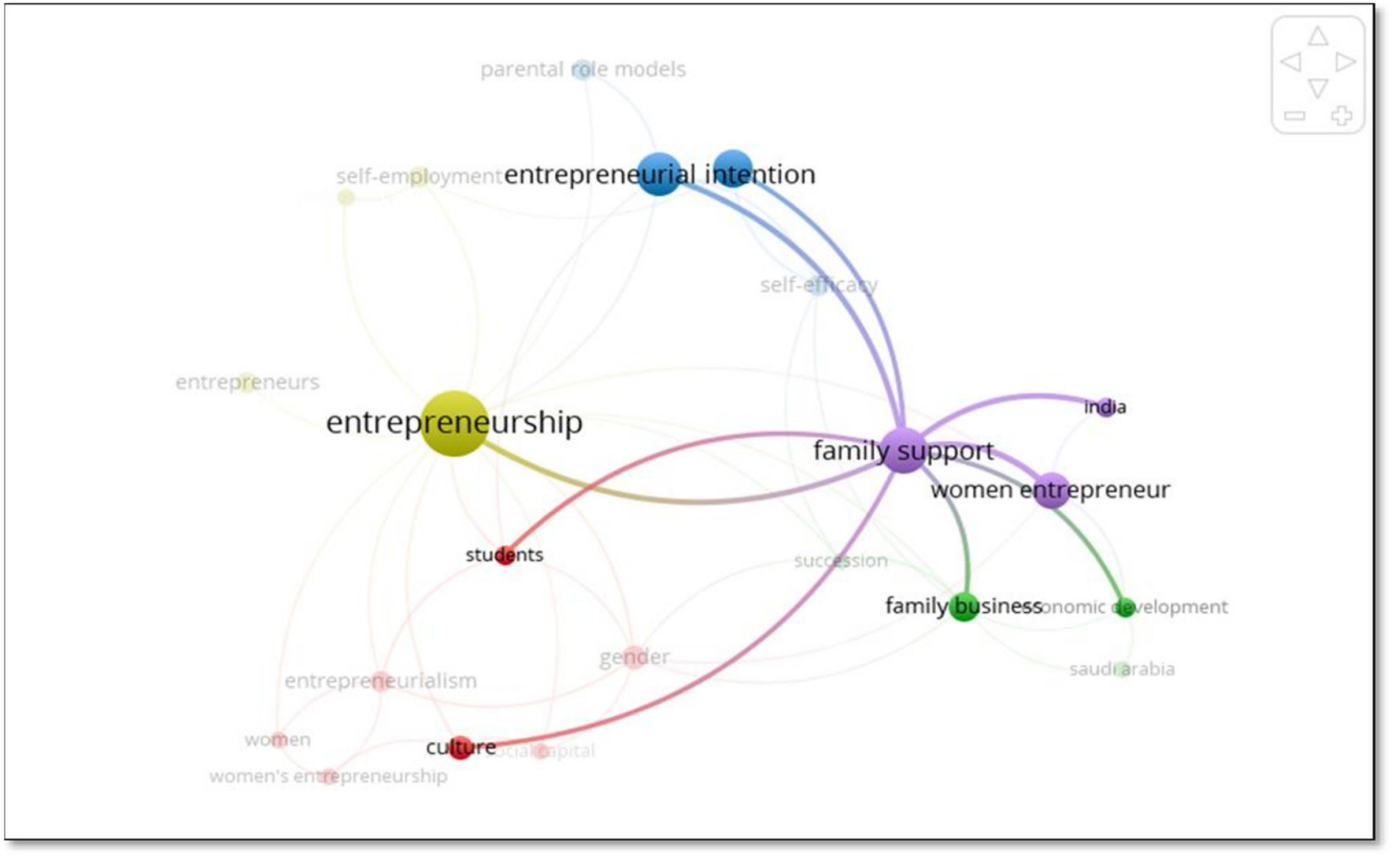
Relations of the violet cluster. Source: VOSviewer version 1.6.10.

Finally, the fifth cluster in purple shows the closeness and strength of connection in the words family support, women entrepreneurs and India. Together with cluster four, it represents the heart of this analysis, which is why even if the number of keywords related to this cluster is low (18.5% of the occurrences) it is the second cluster with the greatest number of relationships with others (Figure 8).

In most societies, especially in developing countries, women's access to entrepreneurship is difficult. The possible explanations have been analyzed in the literature and, although with some socio-cultural differences, they can be summarized as follows: poor social background and lack of support family; conflicts family/care responsibilities; inadequate training; lack of institutional and social interest; consequences of male domination in society and socio-economic discrimination (Kibas, 2006; Mutuku et al., 2006; Lockyer and George, 2012; Raghuvanshi et al., 2017). In recent years, many researchers have analyzed female entrepreneurship and associated limitations (Gautam and Mishra, 2016; Raghuvanshi et al., 2017).

For example, in our analysis, several studies have underlined the importance of family support, when external support systems are limited, especially economically disadvantaged countries or in the case of female entrepreneurship (Pearson et al., 2008; Chang et al., 2009, 2012).

Family support is important with particular reference to women entrepreneurs (Neneh, 2017; Welsh et al., 2018), particularly for those who may not have access to other networks during the business development process (Greve and Salaf, 2003), but also in finding the right balance between family duties and working. In this direction, are the results of a research conducted by Heilbrunn and Davidovitch (2011) with 11 Israeli women entrepreneurs. The support perceived by the family can be even more valuable in the case of entrepreneurial families, because they become models for aspiring entrepreneurs during the process of preparing for the adventure, influencing entrepreneurial intentions (Ahmed et al., 2012; Edelman et al., 2016; Zhu et al., 2017).

As a result, the study also confirmed the positive influence of family members, in terms of support, in the strategic management process. In fact, family members act as positive educational models, which can contribute to starting a business and successful management (Steier, 2003; Arregle et al., 2007), through knowledge and values that are handed down to the children become their human and social capital.

In addition, family members can provide the entrepreneur with a financial start capital of family finances (for example, in the initial phases) or help obtain external funding sources (Aldrich and Cliff, 2003; Anderson et al., 2005). Furthermore, they can offer the necessary work and support that can be useful for creating and managing a business (Teixeira, 2001; Karra et al., 2006).

We could fundamentally highlight two types of family support, emotional/relational, and economic/financial, both a vital resource for supporting entrepreneurship, and useful for both entrepreneurial and economic growth (Shen et al., 2017). In this sense it is wise to expect that emotional support is important especially in developing intentions, as a source of encouragement for those who have no direct experience and can rely on the resources of their families.

While the economic one comes into play, especially in the start-up phase of a business, a transition from intention to behavior, which affects a larger slice of the population.

In line with Aldrich and Cliff (2003), the family plays a key role in the children's enterprise, not only economically, but also by providing knowledge for new initiatives (for example, advice on how to start a business). Sometimes, even “new ideas” (Dyer and Handler, 1994).

## Discussion

Through this work, we carry out a systematic review of the literature on the role of the family in the entrepreneurial process, using different types of bibliometric indicators and cluster analysis.

In the research and selection phase of the articles, we have used various databases of proven utility, such as Scopus, Web of Science and Business Source. Several conclusions emerged from our analysis.

From the results of the bibliometric indicators, it is a relatively recent area of study, but in continuous evolution, considering that the first articles date back to the year 1989, and from a multidisciplinary field of study, which as shown by the analysis of scientific journals, it is mainly linked to the business and management field, and even if in a smaller number, also to social and psychological sciences, economics, and engineering.

Moreover, as shown in Figure 6, research on the role of the family in entrepreneurial activity has grown considerably, especially over the last decade with the United States of America being the country with the most publications on the topic (*n* = 20).

The review also reveals that the scientific journals with the greatest number of publications on the subject is the *International Journal of Entrepreneurial Behavior and Research* (*n* = 6), while the most productive author is Kaciak Eugene (*n* = 4).

Regarding the analysis structure, the most important result is the fact that it is a field of study with non-sharply outlined borders that lacks systematization, probably due to its multidisciplinary character. Indeed, 90.9% of researchers contributed with only one work, this result acquired a greater intensity when it was verified that only 8 authors participated in two or more articles from the examined databases.

As for the cluster analysis, five themes have been highlighted which try to better explain the relationship between family role and entrepreneurship. Specifically, we found: (1) cultural dimension and gender issue, (2) family business and succession, (3) parental role models and entrepreneurial intention, (4) entrepreneurship and self-employment, (5) family support and women entrepreneurs.

Furthermore, the analysis also found that most of the research focused on different themes.

The cluster that obtained the highest percentage of co-occurrences is the yellow one, associated with the following keywords: entrepreneurship, self-employment, entrepreneurs, and personalities, and is also the cluster with the greatest number of relationships with other clusters, especially with family support and exposure to parental role models, emphasizing once again the importance that family has in the entrepreneurial process. On the contrary, the cluster with the lowest percentage of co-occurrences keywords is related to family business, succession, economic development, and Arabia Saudita.

This result could be a good starting point for future research, as it suggests that there are many opportunities to increase and further develop knowledge about the relationship between the role of the family and entrepreneurship. For example, it might be useful to reflect on the possible role that exposure to parental role models plays in corporate succession and analyze any differences through the comparison between entrepreneurial and non-entrepreneurial families. Future research could analyze how and why exposure to models of parental role, or support perceived by family members, has a different influence in different cultures and contexts, especially in disadvantaged contexts, making clear reference to Hofstede's cultural dimensions. It could reflect on why, some contexts, families emotionally support the new generations, promoting entrepreneurial behavior, even in females, while others do not, even if both belong to a stereotypically considered patriarchal culture at the macro level.

Some limitations should be noted. First, in this study, only peer-reviewed articles are considered, eliminating other types of documents, such as book chapters and conference papers. Although this is considered important for the purposes of reliability and quality of the results, it can represent a limit as part of the scientific contributions has been neglected limiting a more detailed knowledge on the research object.

Furthermore, it should be stressed that there is a tendency to mention journals that have open access. There are also journals that can be accessed through payment and that publish articles in languages other than English and Spanish. These are limitations that the reader should consider.

From a purely methodological point of view, some considerations must be made. This article focuses on a group of bibliometric indicators to examine the articles published in the selected databases. Alternative objective analysis techniques and different databases could be useful to provide a systematic description of the literature and to analyze each relevant topic concerning the support of the family from a different point of view, in order to adequately understand the research evolution and propose future research directions in a more accurate way.

Moreover, as regards cluster analysis, even if it is considered a reliable scientific method widely recognized by scholars (Rafols et al., 2010) because it offers an immediate and simple interpretation of the information and the contextualization of a specific one research field, even for non-experts, the boundaries between the various clusters are not always clearly interpreted. This could derive from the fact that the same article can be part of different clusters if it contains keywords that are part of several clusters. For this reason, the mappings should not be considered as tools that provide unequivocal answers to emerging problems, but heuristic methods useful for opening plural perspectives in order to give information about a given field of research.

Furthermore, as pointed out by Rafols et al. (2012), the analysis through maps is very complex in studies on innovation, business, and management as it provides a limited number of significant relationships that take into account the amount of keywords considered (for example, only 22 keywords were generated in this study). This result may be a limitation considering the multidisciplinary nature of the research field and the high fragmentation that characterizes specialized literature.

This study sought to define the boundaries of existing research and at the same time to bring new perspectives of future research, through theoretical and methodological suggestions, aiming to be useful for the development and discovery of new fields of study, expanding the knowledge about the relationship between family support and entrepreneurship. This is an important aspect, not only for academic research and for professionals, but for the agents responsible to promote the entrepreneurial spirit in the community, important as it emerged also in our analysis, at the micro and macro level, for human, social, and economic growth.

## Author Contributions

In the contribution for this survey we describe in detail the following: GC has selected all the useful information for this review. BH-S has provided interesting details on the subject. JS-G examined the final document and the methodological protocol. The authors have decided to approve the final work and take full responsibility for the originality of the research.

### Conflict of Interest

The authors declare that the research was conducted in the absence of any commercial or financial relationships that could be construed as a potential conflict of interest.
