# Supplementary material for: Entrepreneurship and Family Role: A Systematic Review of a Growing Research
Source: Front Psychol. 2020 Jan 10;10:2939. doi: 10.3389/fpsyg.2019.02939 (PMC6967397; doi:10.3389/fpsyg.2019.02939)
Supplement: Supplementary file 1 [file Data_Sheet_1.PDF]

Full list of 92 articles of the Systematic Review

Author(s) and Year	Country	Title	Sample	Design/Approach	Subject Area
Laguía, Moriano & Gorgievski (2019)	Netherlands/Spain	A psychosocial study of self-perceived creativity and entrepreneurial intentions in a sample of university students	559 University Students (58.5% female; 41.5% male)	Quantitative Research	Social Science
Evansluong & Ramírez-Pasillas (2019)	United Kingdom/Sweden	The role of family social capital in immigrants' entrepreneurial opportunity creation processes	Immigrant Entrepreneurs	Qualitative Research	Business and Management
Garcia et al., (2019)	United States	Perceived parental Behaviors and next generation engagement in family firms: A social cognitive perspective		Theoretical Approach	Business
Reay (2019)	United States	Family Routines and Next-Generation Engagement in Family Firms		Theoretical Approach	Business and Management
Eijdenberg et al., (2018)	Netherlands/Germany	Entrepreneurial activities in a developing country: an institutional theory perspective	24 Entrepreneurs	Qualitative Research	Business and Management
Marshall, Dibrell & Eddleston (2018)	United States	What keeps them going? Socio-cognitive entrepreneurial career continuance	247 Business Owners (n=148 female; n=99 male).	Quantitative Research	Business and Management
Al Baghlani (2018)	Iraq	An investigation into strategies used by Iraqi SMEs to survive in the hostile environment: The case of Al-Khaleej company	Case study of AL-KHALEEJ water enterprise in Basra city	Qualitative Research	Business and Management
Lin & Wang (2018)	China	How does the age of serial entrepreneurs influence their re-venture speed after a business failure?	268 Serial Entrepreneurs	Quantitative Research	Business and Management
Lubacha-Sember & Godlewska (2018)	Poland	The role of local formal and informal institutions in microfirms' development: Evidence from Poland	Survey of districts and municipalities n= 46; IDI with MF's owners n= 10; IDI with RCsC n= 2; case study of local law of districts and municipalities n= 52.	Qualitative Research	Social Science
Bignotti & le Roux (2018)	South Africa	Discovering the entrepreneurial endowment of the youth	827 Secondary students	Quantitative Reserch	Economics
Constantinidis et al., (2018)	Belgium/France/Moroco	How families shape women's entrepreneurial sucess in Marocco: an intersectional study	60 Women Entrepreneurs	Qualitative Research	Business and Management

Welsh et al., (2018)	United States/ Canada/ Poland/Morocco	Business-family interface and the performance of women entrepreneurs: the moderating effect economic development	263 Women Entrepreneurs (Morocco: n= 116; Turkey: n= 147).	Quantitative Research	Business and Management
Bretones, & Radrigan (2018)	Spain	Attitudes to entrepreneurship: The case of Chilean and Spanish university students	499 University Students (45.3% from Chile; 54.7% from Spain. N=314 women; n= 185 men)	Quantitative Research	Social Science
Arslan (2018)	Turkish	Fear of failure in entrepreneurs	11 Entrepreneurs	Mixed Method Approach	Business and Management
Pendrian et al., (2018)	Indonesia	Entrepreneurial orientation and strategic initiatives in family business groups: The role of corporate centres and family influence	CEOs and senior management officers in business units	Quantitative Research	Social Sciance
Plopeanu, Homocianu & Airinei (2018)	Romania	Determinants of entrepreneurial proclivity of students in economics and business administration	1.100 University Students	Quantitative Research	Economics
Sher et al., (2017)	Pakistan	An investigation of entrepreneurial intentions of agricultural students	120 University Students	Quantitative Research	Economics
Khan (2017)	Saudi Arabia	Succeeding in challenging environments - female technology start-ups evidence from Saudi Arabia	4 Successful young female technology start-ups	Qualitative Research	Business and Management
Neneh (2017)	South Africa	Family support and performance of women-owned Enterprises: The mediating effect of family-to-work-enrichment	251 Women Entrepreneurs	Quantitative Research	Business and Management
Raghuvanshi, Agrawal & Ghosh (2017)	India	Analysis of barriers to women entrepreneurship: the DEMATEL approach	Women Entrepreneurs	Mixed Method Approach	Business and Management
Zainuddin et al., (2017)	Malaysia	The 21st century challenges among women entrepreneurs: Revisiting the literature	Women Entrepreneurs	Literature Review	Social Science
Shen, Osorio & Settles (2017)	United States	Does family support matter? The influence of support factors on entrepreneurial attitudes and intentions of college students	473 College Students (52% male; 48% female)	Quantitative Research	Business and Management
Batool & Ullah (2017)	China/Pakistan	Successful antecedents of women entrepreneurs: A case of Underdeveloped Nation	Women Entrepreneurs	Quantitative Research	Business and Management
Al-Harthi (2017)	Oman	Understanding entrepreneurship through the experiences of Omani entrepreneurs: Implications for entrepreneurship education	16 Entrepreneurs (50% male; 50% female)	Qualitative Research	Business and Management

Zhu, Burmeister-Lamp & Hsu (2017)	United States/ Netherlands/China	To leave or not? The impact of family support and cognitive appraisals on venture exit intention	Study 1-2: U.S. Entrepreneurs; study 3: Chinese Entrepreneurs.	Quantitative Research	Business and Management
Tiwari & Goel (2017)	India	Success factors of women owned micro and small enterprises in India	Women Entreneurs	Quantitative Research	Business and Management
Ghouse et al., (2017)	United Kingdom	Barriers to rural women entrepreneurs in Oman	10 Women Entrepreneurs	Mixed Method Approach	Business and Management
Munkejord (2017)	Norway	Immigrant entrepreneurship contextualised: Becoming a female migrant entrepreneur in rural Norway	18 Female immigrant entrepreneurs	Qualitative Research	Business and Management
Wahidmurni (2017)	Indonesia	Overcoming business obstacles: A case study of young entrepreneurs in Malang	Young entrepreneurs	Qualitative Research	Business and Management
Khan (2017)	Saudi Arabia	Saudi arabian female startups status quo	80 Women Entrapreneurs	Quantitative Research	Business and Management
Entrialgo & Iglesias (2017)	Spain	Are the Intentions to Entrepreneurship of Men and Women Shaped Differently? the Impact of Entrepreneurial Role-Model Exposure and Entrepreneurship Education	338 final-year undergraduates	Quantitative Research	Business and Management
Kusumojanto et al., (2017)	Indonesia	The effect of entrepreneurship education, parents' role, and self efficacy on students' entrepreneurship intention mediated by entrepreneurship attitudes	347 Students of Economics Faculty	Quantitative Research	Business and Management
Lebdi (2017)	Ireland	The Role of Corporate Parent Support for Spinoff Innovation Performance	Start-up	Quantitative Research	Business and Management
Lee (2017)	Korea	A study on the effect of creativity competency Education on Self-Efficacy and Entrepreneurial Intention: The moderating role of Social support through Parent Cooperation	393 University Students	Quantitative Research	Business and Management
Robin, Astorga & Martinez (2017)	Chile	Entrepreneurial Constraints on Women in Chile: An Empirical Approach	GEM Adult Population Survey data for the period 2011-2015	Quantitative Research	Business and Management
Bose & Mugambi (2017)	India	Entrepreneurial Perceptions Among the Expatriate Graduate Students in the UAE: An Empirical Study	160 Students of engineering and business from three Emirates of UAE (Dubai: n= 39, Sharjah: n= 44 and Ras Al Khaimah: n= 77)	Quantitative Research	Business and Management

Isha, Maneesha & Mafaz (2017)		Key Factors driving innovative Behaviour of women entrepreneurs in Sri Lanka	20 Women Entrepreneurs	Qualitative Research	Economics
Bindah (2017)	Germany	Family dynamics and intergenerational entrepreneurs' leadership style	Entrepreneur Leaders	Quantitative Research	Management
Giacomin, Janssen & Shinnar (2016)	United States/ Belgium/ France	Student entrepreneurial optimism and overconfidance across cultures	1255 University Students (n= 313 American; n= 362 Indian; 580 Spanish) and 353 faculty (n= 79 American; n= 67 Indian and 207 Spanish)	Quantitative Research	Business and Management
Welsh, Kaciak & Thongpapanl (2016)	United States/ Canada/ Poland/Thailand	Influence of stages of economic development on women entrepreneurs' startups	Women Entrepreneurs	Quantitative Research	Business and Management
Wing-Fai (2016)	Ireland	The strenghts of close ties: Taiwanese online entrepreneurship, gender and intersectionality	Nascent entrepreneurs, funders and venture capitaists.	Qualitative Research	Social Science
Soltanian et al., (2016)	Malaysia	Motivations of SME entrepreneurs to become halapreneurs	209 Entrepreneurs SMEs (67,5% men; 32,5% women)	Quantitative Research	Business and Management
Edelman et al., (2016)	United States/Russian Federation	The impact of family support on young entrepreneurs' start-up activities	12.399 Nascent Entrepreneurs	Quantitative Research	Business and Management
Venugopal (2016)	Australia	Investigating women's intentions for entrepreneurial growth	127 Women Entrepreneurs	Mixed Method Approach	Social Science
Mbuya & Schachtebeck (2016)	South Africa	Future entrepreneurs: Does the field of study matter? A comparison of students in a South African urban environment	603 University Students (n= 86 Faculty of Management; n= 517 other Faculties)	Quantitative Research	Business and Management
Guo & Werner (2016)	United States	Gender, family and business: An empirical study of incorporated self-employed individuals in the US	27.147 Entrepreneurs	Quantitative Research	Business and Management
Darmanto & Yuliari (2016)	Indonesia	Motivation, challenges and success factors of woman entrepreneurs in Semarang	180 Women Entrepreneurs	Quantitative Research	Business and Management
Arrighetti et al., (2016)	Italy	Entrepreneurial intention in the time of crisis: a field study	3.684 University Students (58,8% women; 41,2% men)	Quantitative Research	Business and Management
Tarling, Jones & Murphy (2016)	United Kingdom	Influence of early exposure to family business experience on developing entrepreneurs	Students/Graduate	Qualitative Research	Social Science

Rambe & Mokgosi (2016)	South Africa	The influence of personal, family and social variables on technology-oriented venture creation: Theoretical case of internet cafés in Bloemfontein, South Africa		Theoretical Approach	Business and Management
Morales-Alonso, Pablo-Lerchundi & Vargas-Perez (2016)	Spain	An Empirical Study on the Antecedents of Knowledge Intensive Entrepreneurship	851 Engineering Students	Quantitative Research	Business and Management
Nor & Ramli (2016)	Malaysia	A Glimpse at Women Entrepreneurs in Penang	50 Women Entrepreneurs	Quantitative Research	Social Science
Gubik & Farkas (2016)	Hungary	Student Entrepreneurship in Hungary: Selected Results Based on GUESSS Survey	658 Students Entrepreneurs	Quantitative Research	Business and Management
Bignotti & le Roux (2016)	South Africa	Unravelling the conundrum of entrepreneurial intentions, entrepreneurship education, and entrepreneurial characteristics	827 Secondary Students	Quantitative Research	Business and Management
Jabeen, Katsioloudes & Das (2015)	United Arab Emirates/ Qatar	Is family the key? Exploring the motivation and success factor of female Emirati entrepreneurs	224 Emirati female entrepreneurs	Quantitative Research	Business and Management
Obschonka et al., (2015)	Germany/Denmark	Entrepreneurial Self-Identity: Predictors and Effects Within the Theory of Planned Behavior Framework	Entrepreneurs (Sample1,T1: n= 488, T2: n= 200, T3: n= 117; Sample 2, T1: n= 496, T2: n= 205, T3: n= 122)	Quantitative Research	Business and Management
Hoffmann, Junge & Malchow-Møller (2015)	Denmark	Running in the family: parental role models in entrepreneurship	Individual and Parents	Quantitative Research	Business and Management
Goyanes (2015)	Spain	Factors Affecting the Entrepreneurial Intention of Students Pursuing Journalism and Media Studies: Evidence from Spain	310 Students	Quantitative Research	Social Science
Pablo-Lerchundi, Morales-Alonso & González-Tirados (2015)	Spain	Influences of parental occupation on occupational choices and professional values	851 engineering and architecture students	Quantitative Research	Business and Management
Somalingam & Shanthakumari (2015)	India	An Empirical Study on Family’s Business Culture with Reference to Women Entrepreneurs	25 Women Entrepreneurs	Quantitative Research	Business and Management
Welsh et al., (2014)	United States/ Canada/ Poland	Saudi women entrepreneurs: A growing economic segment	164 Women Entrepreneurs	Quantitative Research	Business and Management

Welsh et al., (2014)	United States/ Canada/ Poland	The influence of family moral support and personal problems on firm performance: The case of Korean female entrepreneurs	Female Entrepreneurs	Quantitative Research	Business and Management
Chitra et al., (2014)	India	Does personal factors have an influential role for success of women entrepreneurs in beauty salon industry?	350 Women Entrepreneurs	Quantitative Research	Engineering
Tlaiss (2014)	Canada	Women's entrepreneurship, barriers and culture: insight from the United Arab Emirates	20 Women Entrepreneurs	Qualitative Research	Business and Management
Chahal & Ponnusamy (2014)	India	Study on gender issues in promoting agri-entrepreneurship among farm graduates	70 Graduate Students (50% Male; 50% Female)	Quantitative Research	Management
Pinho & de Sá (2014)	Portugal	Personal characteristics, business relationships and entrepreneurial performance: Some empirical evidence	73 Entrepreneurs (68,5% Male; 31,5% Female)	Quantitative Research	Business and Management
Michael-Tsabari, Labaki & Zachary (2014)	United States/ France/ Israel	Toward the Cluster Model: The Family Firm's Entrepreneurial Behavior Over Generations	Entrepreneurs	Qualitative Research	Business and Management
Amoako & Lyon (2014)	United Kingdom	"We don't deal with": Cooperation and alternative institutions shaping exporting relationships of small and medium-sized enterprises in Ghana	12 exporting SMEs in Ghana	Qualitative Research	Business and Management
Strickland (2013)	Australia	Examining the impact of four key cultural dimensions on ethnic restaurants in Victoria in Australia			Business and Management
Lee & Nyunt (2013)	South Korea	Relationship among the effects of motivations, the critical success and business performance on female - owned businesses: A study of Myanmar female entrepreneurs	Female Entrpreneurs	Quantitative Research	Social Science
Overbeke, Bilimoria & Perelli (2013)	United States	The dearth of daughter successors in family businesses: Gendered norms, blindness to possibility, and invisibility	Successor daughters (n= 7) and sons (n= 6), and non-successor daughters (n= 8) in family businesses	Qualitative Research	Business and Management
Vier Machado, Gazola & Moreno Anez (2013)	Brasil	Creation of enterprises by women: A study with entrepreneurs in Natal, Rio Grande do Norte	96 Women Entrepreneurs	Quantitative Research	Management
Felício, Couto & Caiado (2012)	Portugal	Human capital and social capital in entrepreneurs and managers of small and medium enterprises	Entrepreneurs	Quantitative Research	Business and Management
Pruett (2012)	United States	Entrepreneurship Education: Workshops and Entrepreneurial Intentions	Students	Quantitative Research	Business and Management

Dabic et al., (2012)	United States/ Croatia/ Turkey	Exploring gender differences in attitudes of university students towards entrepreneurship: An international survey	3.420 University Students (63,5% Female; 36,5% Male)	Quantitative Research	Business and Management
Chlosta et al., (2012)	Germany	Parental role models and the decision to become self-employed: The moderating effect of personality	461 University Student (72,8% Male; 27.2% Female)	Quantitative Research	Business and Management
Fritsch & Rusakova (2012)	Germany	Self-employment after socialism: Intergenerational links, entrepreneurial values, and human capital	German households	Quantitative Research	Social Science
Schoon & Duckworth (2012)	United Kingdom	Who becomes an entrepreneur? Early life experiences as predictors of entrepreneurship	6.116 Young People	Quantitative Research	Social Science
Heilbrunn & Davidovitch (2011)	Israel	Juggling family and business: work-family conflict of women entrepreneurs in Israel	111 Women Entrepreneurs (n= 40 Israeli-born women, n= 36 Arab Israeli women and n= 35 women who immigrated from the FSU)	Quantitative Research	Business and Management
Ahmed, Nawaz & Ramzan (2011)	Pakistan	Do external factors influence students' entrepreneurial inclination?	200 Students	Quantitative Research	Economics
Leung (2011)	Canada	Motherhood and entrepreneurship: Gender role identity as a resource	Women Entrepreneurs	Qualitative Research	Social Science
Ozyilmaz (2011)	Turkey	The effects of demographic characteristics on entrepreneurial intention in the pre-venture stage of entrepreneurship	698 University Students	Quantitative Research	Business and Management
Alibaygi & Pouya (2011)	Iran	Socio-demographic determinants of entrepreneurial intentions: A case from Iran	351 rural youth	Quantitative Research	Business and Management
Prasad et al., (2011)	Singapore	Exploring entrepreneurial fulfillmentfor women in India: An empirical study	Women Entrepreneurs	Quantitative Research	Business and Management
Nassif, Andreassi & Simoes (2011)	Brasil	Entrepreneurial Competences: Are there differences between entrepreneurs and intrapreneurs?	Entrepreneurs and Intrapreneurs	Qualitative Research	Management
Chang et al., (2009)	United States/ Canada/ Germany	Family social capital, venture preparedness, and start-up decisions: A study of hispanic entrepreneurs in new England	85 Nascent Entrepreneurs (51% Male; 49% Female)	Quantitative Research	Business and Management
Watkins, Russo & Ochs (2008)	United States	Can students in technology entrepreneurship courses help foster start-ups by the unemployed?	Students and Entrepreneurs	Quantitative Research	Business and Management

Frederick & Foley (2008)	Australia	Does culture and social capital impact on the networking attributes of indigenous entrepreneurs?	Case Study 1: Hawaiian Entrepreneurs; Case Study 2: Australian Indigenous Entrepreneurs; Case Study 3: Maori Entrepreneurs	Qualitative Research	Business and Management
Kickul et al., (2008)	France/ United States	Are misalignments of perceptions and self-efficacy causing gender gaps in entrepreneurial intentions among our nation's teens?	5.000 middle and high school students	Quantitative Research	Business and Management
Carr & Sequeira (2007)	United States	Prior family business exposure as intergenerational influence and entrepreneurial intent: A Theory of Planned Behavior approach	308 individuals from a large southwest U.S. city (members of various ethnic, technology, and small business networking organizations as well as attendees of business start-up seminars within the community)	Quantitative Research	Business and Management
Shivani, Mukherjee & Sharan (2006)	India	Socio-cultural influences on Indian entrepreneurs: The need for appropriate structural inventions	200 Entrepreneurs (n= 150 Male; n= 50 Female)	Quantitative Research	Economics
Scherer et al., (1989)	United States	Role Model Performance Effects on Development of Entrepreneurial Career Preference	Students	Quantitative Research	Management

Note. Elaborated by the Authors
